# Studies of the *in Vitro* Antibacterial Activities of Several Polyphenols against Clinical Isolates of Methicillin-Resistant *Staphylococcus aureus*

**DOI:** 10.3390/molecules190812630

**Published:** 2014-08-19

**Authors:** Yanli Su, Liyan Ma, Yan Wen, Hong Wang, Shuwen Zhang

**Affiliations:** 1 Department of Infection and Critical Care Medicine, Beijing Friendship Hospital, Capital Medical University, Beijing 100050, China; E-Mails: msuer@126.com (Y.S.); mzhuzhu@tom.com (Y.W.); wanghong1812@163.com (H.W.); 2 Clinical Laboratory Center, Beijing Friendship Hospital, Capital Medical University, Beijing 100050, China; E-Mail: ortho_zhu@hotmail.com

**Keywords:** methicillin-resistant *Staphylococcus aureus*, polyphenols, antibacterial activity, synergism

## Abstract

In this study, we report the antibacterial activities of six polyphenols (*i.e*., luteolin, quercetin, scutellarin, apigenin, chlorogenic acid, and resveratrol) against 29 clinical isolates of methicillin-resistant *Staphylococcus aureus* (MRSA), and *in vitro* antibacterial activities of two-drug combinations. All of the MRSA strains evaluated were clinical isolates from patients with MRSA bacteremia. The antibacterial activities were determined by agar dilution method, and the two-drug antibacterial activities were determined by the checkerboard agar dilution method. It was found that luteolin, quercetin and resveratrol show obvious antibacterial activities against MRSA, and the results of two-drug antibacterial activity show either synergy or additivity, without evidences of antagonistic effects.

## 1. Introduction

MRSA (the so called “superbug” as it was originally termed) represents a worldwide threat because of its virulence and broad distribution in community and hospital settings [1]. In many hospitals, MRSA could be detected in over 80 percent of pneumonia sputum samples from severe and elderly patients in the intensive care unit (ICU) [2]. MRSA is the result of the selective pressure of currently used antibiotics, leading to high morbidity and mortality [1]. MRSA normally possesses a multidrug-resistant gene which causes it resistant to β-lactams, aminoglycosides, fluoroquinolones and macrolides [3]. Vancomycin and teicoplanin are the two glycopeptides presently used in clinics for treatment of multi-resistant infections caused by Gram-positive organisms. However, glycopeptide-resistant *S. aureus* (vancomycin-intermediate or resistant *S. aureus*, VISA or VRSA, respectively) are being found with increasing frequency around the World [4]. Therefore, there is an urgent need to develop novel active agents.

**Figure 1:**
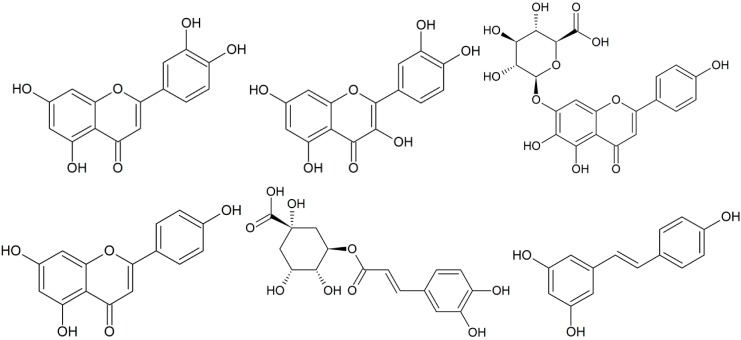
Molecular structures of the six flavonoids luteolin, quercetin, scutellarin, apigenin, chlorogenic acid and resveratrol.

Natural products from higher plants have traditionally been regarded as an important source of antimicrobial agents and have attracted extensive attention in fundamental and clinic applications [5,6]. They are often effective in the treatment of infectious diseases while simultaneously mitigating many of the side effects that are often associated with synthetic antimicrobials [7,8]. Many efforts have been made in the isolation of pure natural products to validate their use in folk medicine and to reveal the active principle(s) [9]. Mixtures of pure phytochemicals as well as various extracts from plants have also been reported to exhibit encouraging antimicrobial activities [7,10,11,12]. Partly inspired by these efforts, there is a keen interest in studying effects of combinations of phytochemicals for antimicrobial applications. Polyphenols from fruits, vegetables and cereals, herbs and spices have shown beneficial effects on human health, and have been found to be effective antimicrobial substances against a wide variety of microorganisms [7,9,13,14,15]. However, to our knowledge, few studies have focused on the synergistic antibacterial activities of combinations of polyphenols. In the present study, we report the antibacterial activities of six polyphenols (luteolin, quercetin, scutellarin, apigenin, chlorogenic acid and resveratrol, Figure 1) against 29 clinical MRSA strains, and their *in vitro in vitro* synergistic activities. These six polyphenols are the main components of a traditional Chinese medicine named as “Compound Qingre Granule”. In our previous study, it was found that this medicine could exhibit antibacterial activities toward MRSA [16]. Due to the aforementioned antibacterial activities of polyphenols, we are very interesting in the antibacterial activities of the polyphenol components of this traditional Chinese medicine toward MRSA. All of these MRSA strains evaluated were clinical isolates from patients with MRSA bacteremia. To estimate antibacterial activities of each polyphenol, a minimum inhibitory concentration (MIC) was determined by the agar dilution method as recommended by the Clinical and Laboratory Standards Institute [17]. Because of poor solubility of these polyphenols in water and Mueller-Hinton broth, studies of antibacterial activities of two-drug combinations were performed by the checkerboard agar dilution method [18] to obtain a fractional inhibitory concentration (FIC) index.

## 2. Results and Discussion

Screening tests of these six different polyphenols were performed against 29 clinical strains of MRSA, one reference strain of MRSA and four strains of methicillin-sensitive *Staphylococcus aureus* (MSSA) using the agar diffusion method. The results are shown in Table 1.

**Table 1:** MICs of six polyphenols against 34 isolates of *S*. *aureus*.

Bacteria		MIC (ug/mL)											
		luteolinn		quercetin		resveratrol		scutellarin		apigenin		chlorogenic acid	
1	MRSA ATCC43300	125		125		1000		>2000		>4000		>4000	
2	SA0922	31.2		62.5		1000		>2000		>4000		>4000	
3	SA0925	62.5		125		1000		>2000		>4000		>4000	
4	SA0927	125		125		500		>2000		>4000		>4000	
5	SA0928	125		125		500		>2000		>4000		>4000	
6	SA0929	125		125		500		>2000		>4000		>4000	
7	SA0930	125		62.5		1000		>2000		>4000		>4000	
8	SA0933	62.5		62.5		500		>2000		>4000		>4000	
9	SA0936	62.5		62.5		500		>2000		>4000		>4000	
10	SA0942	125		62.5		500		>2000		>4000		>4000	
11	SA1032	125		125		1000		>2000		>4000		>4000	
12	SA1039	125		125		1000		>2000		>4000		>4000	
13	SA1040	125		125		1000		>2000		>4000		>4000	
14	SA1053	62.5		31.2		1000		>2000		>4000		>4000	
15	SA1054	125		125		1000		>2000		>4000		>4000	
16	SA1056	62.5		125		250		>2000		>4000		>4000	
17	SA1057	125		125		500		>2000		>4000		>4000	
18	SA1060	125		125		1000		>2000		>4000		>4000	
9	SA1061	125		125		1000		>2000		>4000		>4000	
20	SA1065	31.2		62.5		500		>2000		>4000		>4000	
21	SA1131	125		125		1000		>2000		>4000		>4000	
22	SA1133	125		125		500		>2000		>4000		>4000	
23	SA1134	62.5		125		500		>2000		>4000		>4000	
24	SA1140	62.5		125		500		>2000		>4000		>4000	
25	SA1143	125		125		500		>2000		>4000		>4000	
26	SA1147	62.5		62.5		1000		>2000		>4000		>4000	
27	SA1148	125		62.5		250		>2000		>4000		>4000	
28	SA1154	125		125		500		>2000		>4000		>4000	
29	SA1157	62.5		31.2		500		>2000		>4000		>4000	
30	SA1159	125		125		1000		>2000		>4000		>4000	
1	MSSA ATCC29213	125		125		1000		>2000		>4000		>4000	
2	SA1031	125		125		1000		>2000		>4000		>4000	
3	SA1072	125		125		1000		>2000		>4000		>4000	
4	SA1081	125		125		1000		>2000		>4000		>4000	

It can be seen that the MIC values of luteolin and quercetin for each of these 34 strains range from 31.25 to 125 μg/mL and the MIC values of resveratrol for each of these 34 strains range from 500 to 1000 μg/mL. However, all the MIC values of scutellarin, apigenin and chlorogenic acid are more than 2000 μg/mL. In addition, in the control experiments, no inhibition was observed, indicating that the used solvent dimethyl sulphoxide (DMSO) has no antibacterial activity against either MRSA or MSSA. These results indicate that luteolin, quercetin and resveratrol exhibit remarkable antibacterial activities against MRSA and MSSA, and there are no obvious differences in susceptibility to these three compounds against the MRSA and MSSA strains. On the other hand, scutellarin, apigenin and chlorogenic acid were found to be relatively inactive.

Antibacterial activities of the two-drug combination between the three polyphenol compounds (*i.e*., luteolin, quercetin and resveratrol) were also evaluated by using the checkerboard agar dilution method. Drug interactions are usually classified as synergistic, additive, or antagonistic on the basis of the FIC index. The interaction is defined as: synergy, °0.5; additive effect, 0.5–1; indifference (or no effect), 1–2; antagonism, >2 [19,20,21]. The reductions in the geometric mean (GM) MICs of the drugs when they were given in combination compared to the MICs of the drugs when they were given alone were analyzed by using a paired rank test, which is a nonparametric test for comparison between two related samples. A *p* value of <0.05 is considered significant [19]. When luteolin was combined with quercetin, significant reductions in the GM MICs of luteolin (from 92.56 to 41.20 μg/mL (*p* = 0.000)), and GM MICs of quercetin (from 94.72 to 39.33 μg/mL (*p* = 0.000)) for the MRSA strains were observed. For this combination, synergistic effects were observed in 6.7% (2 of 30) of the interactions. Additive effects were found in 93.3% (28 of 30) of the interactions. No antagonism was observed (see Table 2). When luteolin was combined with resveratrol, significant reductions in the GM MICs of luteolin (from 92.56 to 42.17 μg/mL (*p* = 0.000)), and GM MICs of resveratrol (from 659.75 to 280.62 μg/mL (*p* = 0.000)) for the MRSA strains were observed. For this combination, synergistic effects were observed in 3.3% (1 of 30) of the interactions. Additive effects were found in 96.7% (29 of 30) of the interactions. No antagonism was observed (see Table 3). When quercetin was combined with resveratrol, significant reductions in the GM MICs of quercetin (from 94.72 to 43.16 μg/mL (*p* = 0.000)), and GM MICs of resveratrol (from 659.75 to 274.21 μg/mL (*p* = 0.000)) for the MRSA strains were observed. For this combination, synergistic effects were observed in 3.3% (1 of 30) of the interactions. Additive effects were found in 96.7% (29 of 30) of the interactions. No antagonism was observed (see Table 4). These results show the two-drug combinations between these three polyphenols exhibit either synergy or additivity without evidence of antagonistic effects, which are very encouraging. Owing to the lack of standardization in the methodology used to perform *in vitro* antibacterial susceptibility testing for three or more drugs combinations, we only performed studies of antibacterial activities of two-drug combinations by using the checkerboard agar dilution method.

**Table 2:** FIC indexes of two-drug combination of luteolin-quercetin against 30 MRSA strains.

Bacteria		MIC (μg/mL) of luteolin-quercetin	FIC Index	Interpretation
1	MRSA ATCC43300	62.5/31.2	0.75	ad
2	SA0922	15.6/31.2	1	ad
3	SA0925	31.2/31.2	0.75	ad
4	SA0927	62.5/62.5	1	ad
5	SA0928	62.5/62.5	1	ad
6	SA0929	31.2/31.2	0.5	Syn
7	SA0930	62.5/31.2	1	ad
8	SA0933	31.2/31.2	1	ad
9	SA0936	31.2/31.2	1	ad
10	SA0942	31.2/31.2	0.75	ad
11	SA1032	31.2/62.5	0.75	ad
12	SA1039	62.5/31.2	0.75	ad
13	SA1040	62.5/62.5	1	ad
14	SA1053	31.2/15.6	1	ad
15	SA1054	62.5/31.2	0.75	ad
16	SA1056	15.6/31.2	0.5	syn
17	SA1057	62.5/62.5	1	ad
18	SA1060	62.5/62.5	1	ad
19	SA1061	62.5/31.2	0.75	ad
20	SA1065	15.6/31.2	1	ad
21	SA1131	62.5/62.5	1	ad
22	SA1133	62.5/62.5	1	ad
23	SA1134	31.2/62.5	1	ad
24	SA1140	31.2/62.5	1	ad
25	SA1143	62.5/62.5	1	ad
26	SA1147	31.2/31.2	1	ad
27	SA1148	31.2/31.2	0.75	ad
28	SA1154	62.5/62.5	1	ad
29	SA1157	31.2/15.6	1	ad
30	SA1159	62.5/31.2	0.75	ad

Abbreviations: Syn, synergy; ad, additive effect.

**Table 3:** FIC indexes of two-drug combination of luteolin-resveratrol against 30 MRSA strains.

Bacteria		MIC (μg/mL) of luteolin-resveratrol	FIC Index	Interpretation
1	MRSA ATCC43300	62.5/500	1	ad
2	SA0922	15.6/500	1	ad
3	SA0925	31.2/500	1	ad
4	SA0927	62.5/125	0.75	ad
5	SA0928	62.5/250	1	ad
6	SA0929	62.5/125	0.75	ad
7	SA0930	62.5/500	1	ad
8	SA0933	31.2/250	1	ad
9	SA0936	31.2/250	1	ad
10	SA0942	31.2/250	0.75	ad
11	SA1032	62.5/500	1	ad
12	SA1039	62.5/500	1	ad
13	SA1040	62.5/500	1	ad
14	SA1053	31.2/500	1	ad
15	SA1054	62.5/250	0.75	ad
16	SA1056	15.6/62.5	0.5	Syn
17	SA1057	62.5/125	0.75	ad
18	SA1060	62.5/500	1	ad
19	SA1061	62.5/500	1	ad
20	SA1065	15.6/125	0.75	ad
21	SA1131	62.5/500	1	ad
22	SA1133	62.5/250	1	ad
23	SA1134	31.2/250	1	ad
24	SA1140	31.2/125	1	ad
25	SA1143	31.2/250	0.75	ad
26	SA1147	31.2/500	1	ad
27	SA1148	31.2/125	0.75	ad
28	SA1154	62.5/250	1	ad
29	SA1157	31.2/250	1	ad
30	SA1159	62.5/500	1	ad

Abbreviations: Syn, synergy; ad, additive effect.

**Table 4:** FIC indexes of two-drug combination of quercetin-resveratrol against 30 MRSA strains.

Bacteria			MIC (μg/mL) of quercetin-resveratrol		FIC Index		Interpretation		
1	MRSA ATCC43300		62.5/500		1		ad		
2	SA0922		15.6/500		0.75		ad		
3	SA0925		62.5/500		1		ad		
4	SA0927		62.5/125		0.75		ad		
5	SA0928		62.5/250		1		ad		
6	SA0929		62.5/250		1		ad		
7	SA0930		62.5/500		1		ad		
8	SA0933		31.2/125		0.75		ad		
9	SA0936		31.2/250		1		ad	
10	SA0942		31.2/250		1		ad	
11	SA1032		62.5/500		1		ad	
12	SA1039		62.5/500		1		ad	
13	SA1040		62.5/125		0.75		ad	
14	SA1053		15.6/500		1		ad	
15	SA1054		62.5/250		0.75		ad	
16	SA1056		15.6/62.5		0.375		Syn	
17	SA1057		62.5/125		0.75		ad	
18	SA1060		62.5/500		1		ad	
19	SA1061		62.5/500		1		ad	
20	SA1065		31.2/250		1		ad	
21	SA1131		62.5/500		1		ad	
22	SA1133		62.5/250		1		ad	
23	SA1134		62.5/250		1		ad	
24	SA1140		31.2/250		0.75		ad	
25	SA1143		31.2/250		0.75		ad	
26	SA1147		31.2/500		1		ad	
27	SA1148		31.2/125		1		ad	
28	SA1154		62.5/250		1		ad	
29	SA1157		15.6/250		1		ad	
30	SA1159		62.5/250		0.75		ad	

Abbreviations: Syn, synergy; ad, additive effect.

## 3. Experimental Section

### 3.1. Chemicals

Polyphenols and other chemicals were all purchased from Sigma Chemical Co. (St. Louis, MO, USA). Polyphenols were solubilized in DMSO.

### 3.2. Bacterial Strains

A total of 32 clinical *S. aureus.* strains (MRSA and MSSA) selected from 32 individual patients attending the Beijing Friendship Hospital (Beijing, China) from 2010 to 2012 were studied. All of these isolates were obtained from blood. These strains were identified to the species level by conventional methods (colony morphology, Gram stain characteristics, coagulase reactions). The MRSA strains were defined on the basis of the occurrence of the mecA gene and of their resistance to methicillin and oxacillin, according to the guidelines of the National Committee for Clinical Laboratory Standards (M100-S9). Of 32 strains, 29 strains were mecA-positive, and three strains mecA-negative. ATCC 29213 (a MSSA strain) and ATCC 43300 (a MRSA strain) were used as the reference control strains.

Strains were stored frozen in glycerol broth at −70 °C and subcultured to ensure purity before testing. Test strains, grown overnight at 37 °C in sterile Mueller-Hinton (MH) broth, were resuspended in 0.9% saline to a density equivalent to a 0.5 McFarland standard and then diluted 1:10 in sterile MH broth. MH agar plates containing two-fold serial dilutions of antibacterial agents were inoculated with the final suspensions using an inoculator (Eppendorf Co., Hamburg, Germany) which delivered approximately 10^4^ CFU per spot and incubated at 37 °C for 18–20 h.

### 3.3. Determination of Antibacterial Activity

To estimate the antibacterial activity of individual polyphenol, a MIC was determined by the agar dilution method in MH agar plates, as recommended by the Clinical and Laboratory Standards Institute. Because of poor solubility of these polyphenols in water and MH broth, studies of antibacterial activities of two-drug combinations were performed by the checkerboard agar dilution method to obtain a FIC index. In brief, two-fold serial dilutions of the polyphenol drug were totally solubilized in DMSO, and then were prepared to give initial concentrations four times the MICs of the respective polyphenol alone as determined in individual susceptibility tests. Then, combinations of polyphenol drugs were added with one drug diluted along the x axis and the other drug diluted along the y axis. Thus, for a given range of dilutions every possible combination of drug concentrations was achieved [22]. The media, inocula and conditions were the same as those used for MIC tests. In the control experiments, the same amount of DMSO that was contained in these agents was added into the MH agar to check the antibacterial effect of DMSO in the absence of polyphenols. All experiments were performed in triplicate.

## 4. Conclusions

In conclusion, the results presented in this study have provided useful information on the antibacterial activities of several naturally occurring plant polyphenols against MRSA strains. Luteolin, quercetin and resveratrol exhibit remarkable antibacterial activities against MRSA and MSSA, while scutellarin, apigenin and chlorogenic acid are relatively inactive. Furthermore, the two-drug combinations between luteolin, quercetin and resveratrol exhibit either synergy or additivity without evidence of antagonistic effects, which are very encouraging. Although the lowest values of the MIC exhibited by luteolin or quercetin is relatively higher (31.2 μg/mL) than those of frequently used antibiotics, of which the MIC is generally in the <10 μg/mL range [23], however, the values of the MIC for each polyphenol when used in two-drug combination could drop 2 or 4-fold for all MRSA strains.
